# 

**Published:** 1861-09

**Authors:** 


AMERICAN.

The Pathology and Treatment of Venereal Diseases; including the results of recent investigations upon the subject. By Freeman J. Bumstead, M.D. With illustrations on wood. 8vo., pp. 686. Philadelphia: Blanchard & Lea, 1861.

A Treatise on Diseases of the Joints. By Richard Barwell, F.R.S.C. Illustrated by engravings on wood. 8vo., pp. 463. Philadelphia: Blanchard & Lea, 1861.

A Practical Treatise on the Diseases of the Sexual Organs of Women. By T. M. von Scanzoni. Translated from the French of H. Dor and A. Socien, by A. K. Gardner, M.D. 8vo., pp. 669. New York, 1861.

FOREIGN.

A Manual of Botany; including the Structure, Classification, Properties, and Uses of Plants. By R. Bentley, F.L.S., etc. pp. 811. London, 1861.

On Epilepsy and Epileptiform Seizures; their Causes, Pathology, and Treatment. By E. H. Sieveking, M.D., etc. pp. 336. Second edition, revised and enlarged. London, 1861.

Epileptic and other Convulsive Affections of the Nervous System; their Pathology and Treatment. By C. B. Radcliffe, M.D., etc. pp. 312. Third edition. London, 1861.

Lectures on the Diseases of the Kidney, generally known as "Bright's Disease," and Dropsy. By S. J. Goodfellow, M.D., etc. pp. 306. London, 1861.

Traité Clinique et Pratique des Fractures chez les Enfants. Par Dr. A. Coulon. Revu et précédé d'une Préface par Dr. Marjolin. pp. 264. Paris, 1861.

Traité Pratique de la Pustule Maligne et de l'Œdème Malin, ou des deux formes du Charbon Externe chez l'Homme. Par J. Bourgeois, etc. pp. 316. Paris, 1861.

Die Volkskrankheiten in ihrer Abhängigkeit von den Witterungs-verhältnissen. Von Dr. K. Haller. Wien, 1860.

Ergebnisse und Studien aus der Medicinischen Klinik zu Bonn. Von Dr. M. E. A. Naumann. Band ii. pp. 645. Leipzig, 1860.

On the Relative Influence of Nature and Art in the Cure of Syphilis. By T. Weeden Cooke. pp. 64. London, 1861.

The Physical Examination of the Chest in Pulmonary Consumption and its intercurrent Diseases. By Somerville Scott Alison, M.D., Edinburgh, F.R.C.P., etc. pp. 447. London, 1861.

Clinique Médicale de l'Hôtel-Dieu de Paris. Par A. Trousseau, etc. Tome Premier. pp. 772. 1861.

Why the Shoe Pinches; a Contribution to Applied Anatomy. By Hermann Meyer, M.D., Professor of Anatomy, Zurich. Translated by J. S. Craig, L.R.C.P.E. pp. 55. Edinburgh.

Méthode Pratique du Laryngoscope. Par le Docteur L. Türck, of Vienna. pp. 124. Edition Françaisе. 1861.

The Modern Pathology and Treatment of Venereal Diseases. By P. H. Watson, M.D., F.R.C.S. pp. 39. Edinburgh and London, 1861.

Obstetric Aphorisms, for the Use of Students commencing Midwifery Practice. By J. G. Swayne, M.D., etc. pp. 130. Second edition. London, 1861.

Ready Rules for Operations in Surgery. By A. Webb, M.D., Surgeon-Superintendent to Native Hospital, Calcutta. pp. 98. London.

The Autobiography and Services of Sir James McGrigor, Bart., late Director-General of the Army Medical Department. With an Appendix of Notes and General Correspondence. pp. 418. London, 1861.

